# Phytochemical Profile, Pharmacological Attributes and Medicinal Properties of *Convolvulus prostratus* – A Cognitive Enhancer Herb for the Management of Neurodegenerative Etiologies

**DOI:** 10.3389/fphar.2020.00171

**Published:** 2020-03-03

**Authors:** Acharya Balkrishna, Pallavi Thakur, Anurag Varshney

**Affiliations:** ^1^ Drug Discovery and Development Division, Patanjali Research Institute, Haridwar, India; ^2^ Department of Allied and Applied Sciences, University of Patanjali, Haridwar, India

**Keywords:** *Convolvulus prostratus*, *Shankhpushpi*, natural product, Ayurveda, neuroprotective, nootropic

## Abstract

*Convolvulus prostratus* Forssk., a nootropic herb used in traditional medicinal systems, is also frequently known by its taxonomic synonym *Convolvulus pluricaulis*. In Indian medicinal system – *Ayurveda* – it is named as *Shankhpushpi*. According to the ancient literature, this herb has been attributed with several therapeutic properties, such as anxiolytic, neuroprotective, antioxidant, analgesic, immunomodulatory, antimicrobial, antidiabetic and cardioprotective activities. This medicinal herb has been reported to contain many bioactive phytoconstituents, such as, alkaloid (convolamine), flavonoid (kaempferol) and phenolics (scopoletin, β-sitosterol and ceryl alcohol), that have been ascribed to the observed medicinal properties. Several research teams across the globe have highlighted the neuro-pharmacological profile of *C. prostratus*, wherein, the neuroprotective, nootropic and neuro-modulatory roles have been described. Besides, role of *C. prostratus* extracts in neurodegeneration has been well demonstrated. Despite of such elaborative preclinical pharmacological profile, detailed clinical investigations and mechanistic mode-of-action studies of this important herb are yet to be executed. The present review is attempted to showcase the phytochemical profile, pharmacological attributes and medicinal information of *C. prostratus*; with comprehensive research gap analysis. It is hoped that the scientific update on the ethnomedicinal aspects of this herb would thrive research propagation and development of the CNS phytopharmaceuticals, originated from *C. prostratus*.

## Introduction

In recent years, non-communicable diseases (NCDs) have become an emerging cause of morbidity and mortality (~ 12% global prevalence) (Islam et al., 2014). Neurological disorders constitute a significant proportion as the leading causes of death among all the other non-communicable diseases (Gourie-Devi, 2014). The most common disorders of the nervous system are schizophrenia (~ 40% prevalence in India) and epilepsy (~ 45% prevalence in India), which are clinically presented by dysfunction of the interneurons, misbalancing neuronal homeostasis, ultimately leading to neurophysiological disintegration (Gururaj et al., 2005). It is estimated that by next decade, mental and behavioural disorders will lead to a reduction in the average life expectancy, nearly by one-fifth proportion (Lopez and Murray, 1998). In India, an average of 65 people out of 1,000 inhabitants suffer from mental and behavioural disorders, wherein, maximum prevalence has been recorded for alcohol dependency afflicted mental disorders (~ 6% prevalence), child and adolescent behavioural disorders (~ 4.3% prevalence) and mood disorders (~ 1.6% prevalence). These statistics makes it obvious that the prevalence of neurodegenerative disorders is escalating, however, effective and safe treatment modalities are still under infancy (Gururaj et al., 2005).

The chemotherapeutic moieties used currently for the treatment of neurological disorders face a daunting challenge of systemic delivery of the drugs to the central nervous system. Moreover, crossing the blood brain barrier after strenuous systemic administration is another challenge. Ultimately, the bioavailability of these drugs becomes low and therefore, lead to below- optimal efficacies (Tonda-Turo et al., 2018). Several drug delivery vehicles and nano-formulations have been devised so as to facilitate the targeted delivery of the drug to the site of action, *i.e.*, the central nervous system, thereby bypassing the blood brain barrier (Saraiva et al., 2016; Tonda-Turo et al., 2018). However, these strategies have also shown little success. Most of the neurodegenerative disorders are progressive in nature, and the cycle of etiological events usually have an early-onset which might get unnoticed or under-detected. In such a case, the therapies initiated after the onset of neuropathological asymptomatic etiologies will only have limited value for the patients (Solanki et al., 2016). Moreover, the foresaid neuropathological conditions are manifested with several additional etiologies, wherein the chemotherapeutic modalities act typically on a single target, thereby providing only palliative care (Chen and Pan, 2014). Hence, natural compounds serve as the holistic option which act on multiple neural targets and are enriched with free radical scavenging polyphenolic compounds (Park et al., 2018). These phytoconstituents have well-described antioxidant and anti-inflammatory properties which aids in protecting the neuronal cells against oxidative stress. Additionally, the natural compounds, such as flavonoids also aid in modulating the neuronal cell signalling pathways (Solanki et al., 2016).

There are various herbs which are used in traditional medicine for the management of neurodegenerative diseases, for instance: *Centella asiatica* (L.) Urb., *Glycyrrhiza glabra* L., *Tinospora cordifolia* (Willd.) Hook. f. & Thomson, *Bacopa monnieri* (L.) Wettst. and *Nardostachys jatamansi* (D. Don) DC. (Kulkarni et al., 2012). One such example is *Convolvulus prostratus* Forssk. (Syn. *Convolvulus pluricaulis* Choisy), which is commonly known as *Shankhpushpi* in *Ayurveda* and is widely recognized for its anxiolytic, antidepressant and nootropic activities (Kulkarni et al., 2012). As per an ancient Indian medicinal scripture—*Charaka Samhita*—this plant is superior to other nootropic drugs (*Medhya rasayana*) of *Ayurveda*, however, a detailed ethnomedicinal update yet needs to be presented (Dev, 2006). The ensuing sections will provide an ethnomedicinal, phytochemical and pharmacological update of this cognitive booster herb, *C. prostratus* (CP).

## Ethnomedicinal Update on *C. prostratus*

**Figure 1:**
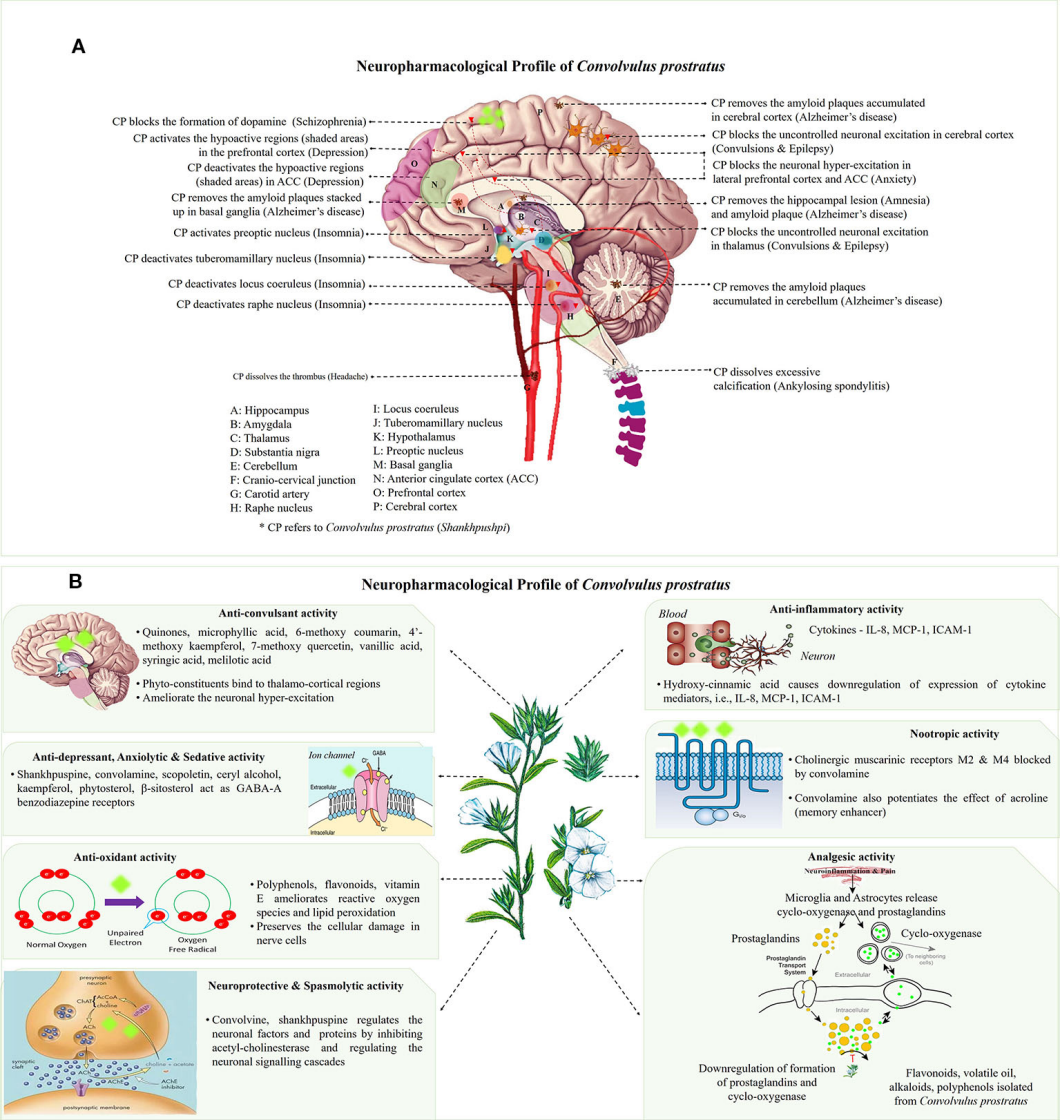
**(A)** Neuro-mechanistic aspects of *Convolvulus prostratus* (CP). Schematic representations of *C. prostratus* (CP) target and mode of action in neural system. CP reduces the lesions formed in the hippocampus (A); and amyloid plaque accumulation in hippocampus (A), cortex (P), basal ganglia (M) and cerebellum (E). CP also acts as GABA-A agonists and binds to lateral prefrontal cortex (O) and anterior cingulate cortex (N). CP deactivates the wake promoting areas, namely, tuberomamillary nucleus (J) located in the hypothalamus (K), locus coeruleus (I) and raphe nucleus (H). It also activates the sleep inducing areas of the brain, namely, preoptic nucleus (L). Moreover, CP activates the hypoactive regions situated in the prefrontal cortex (shaded pink area; O), and deactivates hyperactive regions situated in the anterior cingulate cortex (shaded green area; N). CP blocks the excessive production of dopamine as produced *via* substantia nigra (D). CP also removes the excessive calcification formed at the cranio-cervical junction (F). Additionally, CP removes the formation of thrombus in the carotid artery (G). **(B)** Main mechanisms and phytoconstituents responsible for neuro-pharmacological activities of *C. prostratus* (CP). This herb exhibits anti-convulsant, anti-depressant, anxiolytic, sedative, anti-inflammatory, anti-oxidant, analgesic, nootropic, spasmolytic and neuroprotective activities. Phyto-constituents belonging to diverse chemical families, namely, coumarins, alkaloids and polyphenols (see **Table 2**) are responsible for such evident neuro-pharmacological profile of the CP plant (Nolte, 1999; Carter, 2014). Reported mechanisms of CNS action of CP phyto-constituents has been depicted in these schematics, with putative site of action has been marked with fluorescent green blobs.

*C. prostratus* belongs to Convolvulaceae family and is ubiquitous in the north-western regions of India (Nisar et al., 2012). In *Ayurveda*, this herb is classically described as a memory and intellect booster. Moreover, it is employed in a variety of formulations used for the treatment of nervous disorders, such as insanity, epilepsy, hysteria, insomnia, and psycho-neurosis (Khare, 2004). Mechanistically, it reduces the spontaneous motor activity, thereby controlling the refluxes and frightening responses. It ultimately acts as a sedative moiety which initiates a persistent fall in blood pressure and cardiac contraction, thereby managing neurological pathologies, such as anxiety, insanity and epilepsy (Chaudhary, 1996). The neuro-mechanistic aspects of this herb has been elucidated in **Figure 1A**. Besides, this plant has manifold therapeutic utilities, wherein, a decoction of its shoots is used as a remedy for anaemia and weakness (Singh et al., 2003). More specifically, in ancient texts, this plant has been mentioned as *sara*, *medhya*, *vrsya* and *rasayana*, which refers to the laxative, nootropic, aphrodisiac and rejuvenator properties of this herb, respectively. Additionally, one of the revered ancient Indian medical practitioner, *Acharya Charaka* had used white-flowered variety of *C. prostratus* (*Shankhpushpi*) along with the juice of *Bacopa monnieri* (*Brahmi*), *Acorus calamus* (*Vacha*) and *Saussurea lappa* (*Kushtha*) for alleviating insanity and epilepsy. Similar views had been presented in *Chikitsasangraha* written by *Chakradatta*; *Kaideva Nighantu*; and *Ayurveda Saar Sangraha* (Khare, 2004).

Besides *Ayurveda*, *C. prostratus* (CP) has also been used in *Siddha* system of medicine, wherein an oil obtained from this plant is used as a keratogenic agent for promoting hair growth (Gogte, 2012). It is also believed that a paste prepared from its roots and flowers act as anti-aging agents, thereby indicating its apparent anti-oxidant activity (Adams et al., 2007). Furthermore, in *Unani* medicinal system, a syrup prepared with *C. prostratus* and *Piper nigrum* is prescribed in bleeding piles and venereal diseases (Khare, 2004). All the above mentioned ethnomedicinal uses of *C. prostratus* have been tabulated in **Table 1**.

**Table 1:** Ethnomedicinal uses of *Convolvulus prostratus* (CP) with predominant plant parts and mode of herbal preparation.

Disease Targeted	Part(s) used	Method of Preparation and Dosage (if any)
Amnesia^†^	Whole plant	Decoction of this herb used along with milk (Bhalerao et al., 2014).
Anorexia	Leaf	Intact part used as such (Rizwan and Khan, 2014).
Anxiety^†^	Leaf, Flower	Intact parts used for treating anxiety neurosis (Mishra and Sethiya, 2010).
Arthritis	Whole plant	Whole plant used for the management of arthritis, osteoarthritis and rheumatic pain (Pan et al., 2009).
Asthma	Leaf	Intact part used as such (Mishra and Sethiya, 2010).
Blood disorders	Whole plant	Whole plant is used (Nisar et al., 2012).
Bone fracture	Leaf, Flower	Paste of flowers and leaves used for the management of fracture (Singh and Pandey, 1998).
Bronchitis	Leaf	Intact part used as such (Mishra and Sethiya, 2010).
Burning sensation^†^	Whole plant	Whole plant is used (Sethiya et al., 2009).
Calculi	Leaf	Intact part used as such (Mishra and Sethiya, 2010).
Constipation	Leaf, Stem	Intact parts used for the management of constipation (Malik et al., 2015).
Cough	Leaf	Intact part used as such (Rizwan and Khan, 2014).
Dementia^†^	Root, Flower	Paste of roots and flowers administered for the treatment of dementia (Adams et al., 2007).
Diabetes	Leaf	Paste of leaves (100 g) along with black pepper (3-4 grains) is to be administered once daily (Singh et al., 2003).
Dyspnoea	Leaf	Intact part used as such (Rizwan and Khan, 2014).
Dysuria	Whole plant	Jelly obtained from this plant (10 g) is mixed with honey (10 g) and is to be taken thrice a day (Bhutya, 2011).
Edema	Whole plant	Whole plant is used (Sethiya et al., 2009).
Emesis	Whole plant	Whole plant is used (Agarwa et al., 2014).
Enuresis	Whole plant	Powder of this plant along with *Hyoscyamus niger*, *Prunella vulgaris*, (l-2 g) and milk (100 mL) is to be taken along with twice a day (Bhutya, 2011).
Epilepsy^†^	Whole plant	Paste of this plant along with cumin seeds (l g) and milk thrice a day (Bhutya, 2011).
Gonorrhoea	Whole plant	Jelly obtained from this plant (10 g) is mixed with honey (10 g) and is to be taken thrice a day (Bhutya, 2011).
Haemoptysis	Leaf	Juice of the leaves has to be administered at a dose of 10 mL, thrice a day (Bhutya, 2011).
Haemorrhoid	Whole plant	Whole plant is used (Nisar et al., 2012).
Headache^†^	Whole plant	Powder of plant (3 g) mixed with sugar (5 g) and milk (20 ml) is to be taken twice daily (Bhutya, 2011).
Hematemesis	Whole plant	Whole plant is used (Dhingra and Valecha, 2007).
Hysteria^†^	Whole plant	Intact plant is used (Dhingra and Valecha, 2007).
Insomnia^†^	Whole plant	Whole plant is used (Dhingra and Valecha, 2007).
Leprosy	Whole plant	Whole plant is used (Nisar et al., 2012).
Leucoderma	Whole plant	Whole plant is used (Bhutya, 2011).
Menorrhagia	Whole plant	Paste of this plant is taken along with milk (Gupta, 2016).
Neurological disorders^†^	Whole plant	Decoction of this herb is used along with cumin and milk for treating attention deficit hyperactivity disorder (ADHD), mild convulsions, depression, emotional stress, mental debility, memory loss, mental hypersensitivity, schizophrenia and stress disorders (Mishra and Sethiya, 2010; Sultana et al., 2018).
Polydipsia	Leaf, Stem	Intact parts used for the management of excessive thirst (Malik et al., 2015).
Pyrexia	Root, Leaf	Intact parts used for alleviating fever (Bhalerao et al., 2014).
Pyrosis	Leaf, Stem	Intact parts used for the management of heart burn or pyrosis (Malik et al., 2015).
Scrofula	Whole plant	Decoction of this herb is used along with cumin and milk (Mishra and Sethiya, 2010).
Sexual debility	Whole plant	This plant (100 g) is ground with little water and taken with sugar or honey once daily, for 21 days (Bhutya, 2011).
Snake bite	Whole plant	Whole plant is used (Sethiya et al., 2009).
Stomachache	Whole plant	One tea spoon powder of dried plant is taken for the management of stomachache (Katewa and Jain, 2006).
Syphilis	Whole plant	Decoction of this herb is used along with cumin and milk (Mishra and Sethiya, 2010).
Ulcer	Root	Intact part used as such (Bhutya, 2011).
Urinary diseases	Leaf	Intact part used as such (Rizwan and Khan, 2014).
Vertigo^†^	Whole plant	A syrup prepared from this herb is used for the management of vertigo (Gogte, 2012).
Worm infestation	Whole plant	Whole plant is used (Gupta, 2016).
Wound	Whole plant	Whole plant is used (Trivedi, 2009).

^†^Shaded rows indicate specific neurological diseases which can be managed by the administration of C. prostratus.

## Industrial Significance of *C. prostratus*

*C. prostratus* is extensively used in pharmaceutical, cosmeceutical and nutraceutical industries (Jalwal et al., 2016). In the pharmaceutical industry, various extracts, syrups and tablets are produced, specifically for targeting neurodegenerative diseases, hypertension, hypercholesterolemia and gastric ulcers. A few examples of such marketed herbal pharmaceutical formulations include Patanjali Divya Shankhpushpi Churna™, Divya Pharmacy Shankhpushpi Sharbat™, Baidyanath Shankhpushpi Sharbat™, Dabur Shankhpushpi Syrup™, Biotrex Shankhpushpi™, Maxmind capsule™, Herbal Hills Shankhpushpi Tablets™ and many more (Bhowmik et al., 2012). Similarly, in the cosmeceutical industry, the CP herb is used as a general tonic for rejuvenating the skin and hairs, thereby treating skin related ailments as well as keratogenic disorders. A few examples for elucidating the use of CP (Shankhpushpi) as cosmeceutical ingredients include Econature Shankhpushpi Hair oil™, Khadi Natural Shankhpushpi oil™ and Alps Shankhpushpi hair Mask Powder™. Moreover, the CP powder and juice, such as, Jain Shankhpushpi Powder™ and Axiom Jeevan Ras Shankhpushpi Juice™, are also being used as skin mask for rejuvenating the skin and managing skin problems such as acne, blemishes and sun spots (Hindu, 2012). Additionally, food grade CP powder and syrups are also available in the market for being used as a nutraceutical nootropic supplement, for example, Divya Pharmacy Shankhpushpi Sharbat™, Baidyanath Shankhpushpi Sharbat™, Shivalik Herbals Shankhpushpi Nutraceutical Capsules™ and Veg E Wagon *Shankhpushpi* Powder (Bhowmik et al., 2012). Such extensive industrial uses of CP further confirms the holistic significance of this nontoxic wonder herb (Jalwal et al., 2016).

## Phytomedicinal Formulations Containing *C. prostratus*

The CP herb has also been used as a phyto-ingredient of a polyherbal medicinal formulation: *Sankhahauli*, which contains leaves of *C. prostratus* (15 *g*); seeds of *Piper nigrum* (3 *g*) and *Papaver somniferum* (20 *g*); whole plant of *Prunus amygdalus* (10 g), *Vitis vinifera* (20 g) and *Coriandrum sativum* (10 *g*). This formulation is mainly used for the management of insomnia, drug addiction and hypertension (Bhutya, 2011). Several such marketed herbal formulations containing CP are being used for the management of a variety of neurological ailments in India, for example., *Divya Medha Vati*, *Divya Medha Kwath* (Patanjali Ayurved Ltd.); BR-16A (Himalaya Drug Co. Ltd.); *Dimagheen* (Dawakhana Tibiya College); *Shankhpushpi* syrup (Unjha); *Shankhavali Churna* (Narnaryan Pharmacy); Brain tab and *Shankhpushpi* syrup (Baidyanath Pharmaceuticals) (Aggarwal et al., 2011; Sethiya et al., 2013; Amin et al., 2014).

## Chemical Profile of *C. prostratus*

All of these stated medicinal utilities of *C*. *prostratus* (CP) have been attributed to various phytoconstituents, belonging to the chemical family of alkaloids, flavonoids, coumarins and polyphenols. Among these phytoconstituents, certain compounds are known to be present at a higher concentration (almost 20% w/w) and are known as major phyto-constituents. CP plant is known to contain kaempferol, β-sitosterol, N-hexacosanol, taraxerol, taraxerone, delphinidine and hydroxy-cinnamic acid as the major phyto-constituents, as depicted in **Table 2** (Billore et al., 2005; Amin et al., 2014). Moreover, an alkaloid, namely, Sankhpuspine has also been isolated from this plant and is known as a chemotaxonomic marker for this species (Basu and Dandiya, 1948; Saroya and Singh, 2018). CP plant also contains other alkaloids (convolamine, convosine, convoline, convolidine, convolvine, confoline, evolvine, phyllabine, subhirsine, sankhpuspine) (Agarwa et al., 2014; Balaji et al., 2014); anthroquinones; carbohydrates (D-glucose, sucrose, rhamnose, maltose) (Dhingra and Valecha, 2007; Bhowmik et al., 2012; Agarwa et al., 2014); coumarins (ayapanin, scopolin, scopoletin); flavonoids (kaempferol, quercetin) (Lal, 2014); glycosides (geranilan-3-ol-1-carboxylate-1-O-β-D-xylopyranosyl-(2′→1′′)-O-β-D-xylopyranoside (Sultana et al., 2018); phenolic compounds; steroids; tannins; and terpenoids (Ravichandra et al., 2013; Agarwa et al., 2014; Balaji et al., 2014; Malik et al., 2016).

**Table 2:** Major phyto-constituents of *Convolvulus prostratus* (CP) with their reported medicinal utility.

Name of the compound	Category of phytoconstituent	Chemical structure^†^	Medicinal profile
**Ayapanin**	Coumarin	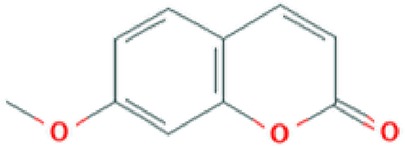	Improves scopolamine-induced spatial memory impairment (Zingue et al., 2018); anti-nociceptive activity (Cheriyan Sr et al., 2017).
**Convolamine**	Alkaloid	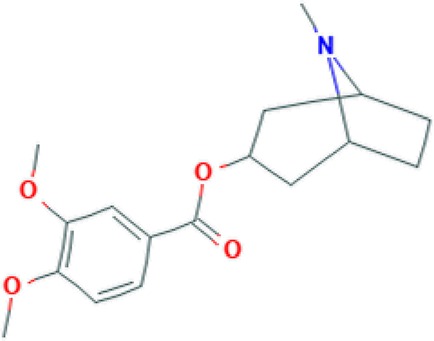	Antihypoxic, immune-modulating, and anti-inflammatory activity (Gapparov et al., 2011).
**Convoline**	Alkaloid	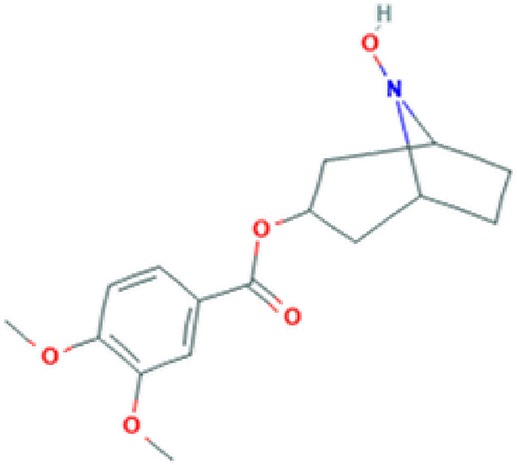	Anti-epileptic activity (Dubey et al., 2018).
**Convolvine**	Alkaloid	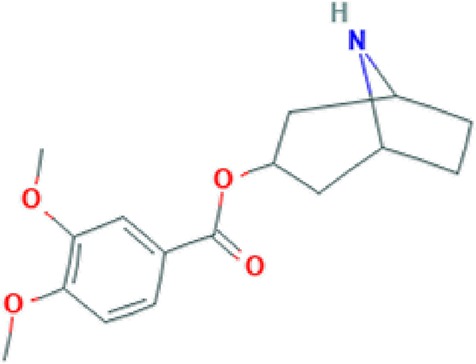	Antihypoxic, immune-modulating, and anti-inflammatory activity (Gapparov et al., 2011); blocks the M-receptors; exhibits nootropic, cytotoxic and sedative activity (Mirzaev and Aripova, 1998; Tseomashko et al., 2013).
**Delphinidine**	Anthocyanin	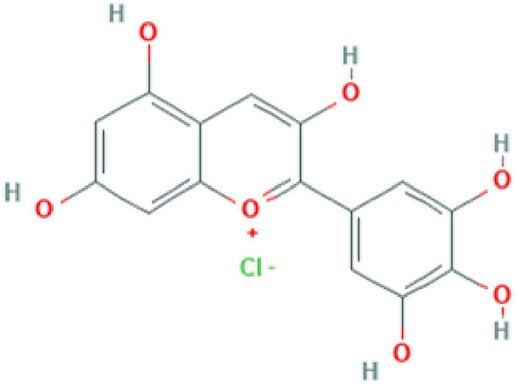	Antioxidant, anti-mutagenic, anti-inflammatory and antiangiogenic (Patel et al., 2013).
**Hydroxy-cinnamic acid**	Carboxylic acid	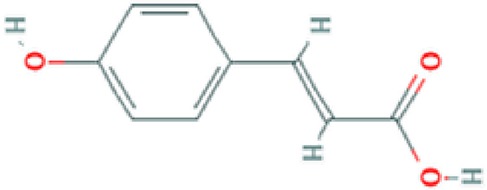	Antioxidant, photooxidant activity; strong inhibitory effect on the tumour U14 (Vinholes et al., 2015).
**Kaempferol**	Flavonoid	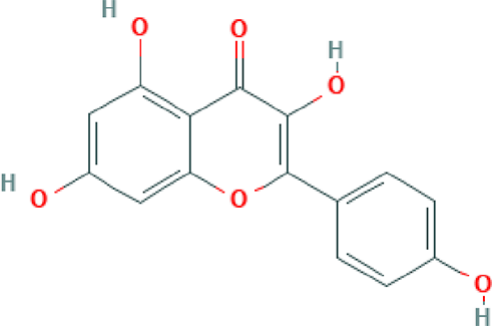	Activates LXR-β and suppresses SREBP-1 to enhance symptoms in metabolic syndromes; potent inhibitory effect on *in vitro* bone resorption; anti-inflammatory, anti-oxidant activity; inhibition of cancer cell invasion through blocking the PKCδ/MAPK/AP-1 (Wang et al., 2015; Hoang et al., 2015).
**Quercetin**	Flavonoid	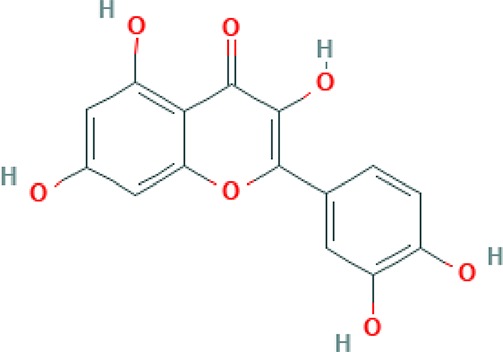	Antioxidant activity; stimulator of recombinant SIRT1 and also a PI3K inhibitor; attenuated the function VEGFR, androgen receptor and the expressions of NF-κB, IL Receptor, FAK, ERK, Nrf2 (Xing et al., 2001).
**Scopoletin**	Coumarin	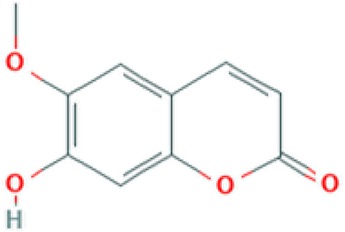	Antifungal, anti-allergic, anti-aging and hypouricemic activities (Sun et al., 2014; Nam and Kim, 2015).
**Scopolin**	Coumarin glucoside	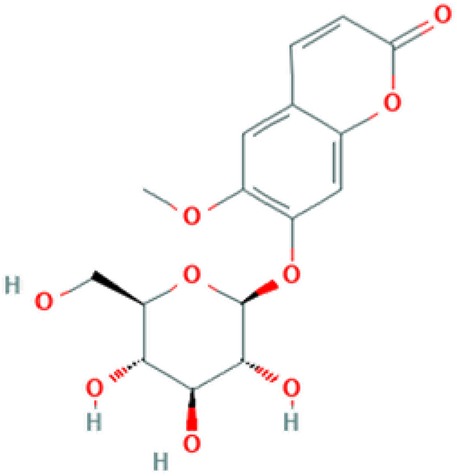	Antinociceptive activity; acetylcholinesterase (AChE) inhibitor; fungitoxic activity (Prats et al., 2006; Pan et al., 2009).
**Taraxerol**	Triterpenoid	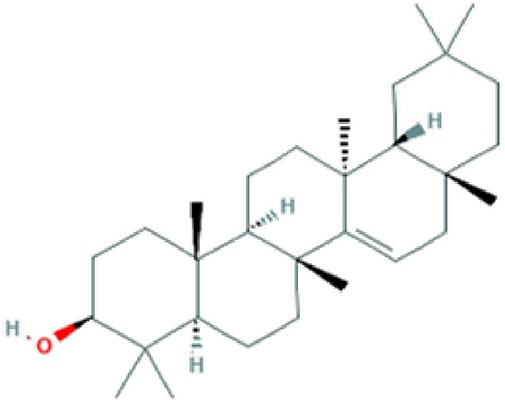	Anti-inflammatory; anti-cancerous activity (Swain et al., 2012).
**β-sitosterol**	Phytosterol	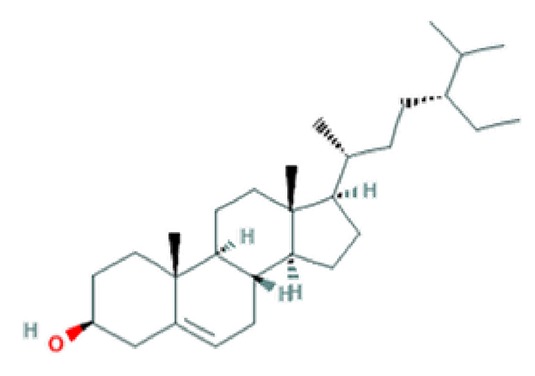	Anti-inflammatory; anti-proliferation; anti-pyretic; pro-apoptotic activity (Gupta et al., 1980; Park et al., 2007).

^†^Source of Chemical structures: PubChem (https://pubchem.ncbi.nlm.nih.gov/).

Several other hydrocarbons, namely, 1- pentyl-2-tridecanyl cyclopentyl cyclohexane carboxylate, 1,2-benzenedicarboxylic acid, 10-bromodecanoic acid, 1-octadecanesulphonyl chloride, 2-butanone, 2-pentanol, 7- hydroxyheptadecanyl-1,7, 17-tricarboxylic acid, ascorbic acid, cyclononasiloxane, cyclo-octadecanyl methanol, decanoic acid, dicyclohexyl cyclo-octyl acetic acid, eicosane, heneicosane, hydroxy cinnamic acid, octatriacontyl pentafluoropropionate, pentadecyl 2-propyl ester, pentanoic acid, pentyl hexacosanoate, phthalic acid, silane, squalene, tetracyclohexanyl caproate and tridecane are also found in the extract of the CP plant (Bhalerao et al., 2014; Malik et al., 2016; Rachitha et al., 2018; Sultana et al., 2018).

In addition, CP is known to be a good source of vitamins and minerals, namely, calcium, copper, iron, magnesium, manganese, phosphorus, potassium, sulphur, zinc, vitamin C and E (Sethiya et al., 2010; Babu et al., 2015).

## Neuro-Pharmacological Profile of *C. prostratus*

### Nootropic Activity

*C. prostratus* (CP) contains volatile oil; fatty alcohols; flavonoids, *i.e.* kaempferol; hydroxy cinnamic acid; β-sitosterol; and carbohydrates such as glucose, rhamnose, sucrose *etc.*, which endow this plant with nootropic capabilities. Moreover, an alkaloid, namely, convolvine present in this herb has also been found to block cholinergic muscarinic receptors: M2 and M4. Convolvine also aids in potentiating the effect of another muscarinic memory enhancer, namely, arecoline, thereby imparting nootropic abilities to CP (Sethiya et al., 2009). In a study, Rawat and Kothiyal have also found that the aquo-methanolic, ethanolic and petroleum ether extracts isolated from CP (50–400 mg/Kg) exhibited anxiolytic, memory-enhancing and nootropic activity as evaluated by using Elevated Plus Maze (EPM) and step-down models in mice. EPM test has mainly been used to investigate the interactions between aversive memory and anxiety responses of the mice. CP effects on EPM activity has been found to be comparable to the standard of care drug, Piracetam (Rawat and Kothiyal, 2011; Kaushik, 2017). Moreover, treatment with alcoholic extract of CP plant led to an increase in the average time-span spent by mice in the enclosed arm of plus maze model, and an escalation in the mean avoidance response on the jumping box model (Rawat and Kothiyal, 2011).

### Neuroprotective Activity

The aqueous extract of the roots of *C. prostratus* inhibited the activity of acetylcholinesterase (AChE) within the cortex and hippocampus of male Wistar rats, that have been intoxicated with scopolamine. CP extract also posed evident anti-oxidant activity and elevated the levels of glutathione reductase, superoxide dismutase and reduced glutathione within the cortex and hippocampus (Kaushik, 2017). Similar results have been observed in case of aluminium chloride induced neurotoxicity in rat cerebral cortex. Regular administration of the CP root extracts (150 mg/Kg) for 3 months inhibited the decline in Na^+^/K^+^ ATPase activity and also preserved the mRNA expression levels of muscarinic acetylcholine receptor 1 (M1 receptor), choline acetyl transferase (ChAT) and nerve growth factor-tyrosine kinase A receptor (NGF-TrkA) (Bihaqi et al., 2009; Kaushik, 2017). The Na^+^/K^+^ ATPase pump aids in maintaining the osmotic equilibrium and membrane potential in neuronal cells (Forrest, 2014). Secondly, the muscarinic receptors bind to the neurotransmitter acetylcholine, thereby facilitating the transmission of electrical signals within the central nervous system (Brown, 2018). Furthermore, the choline acetyl transferase enzyme is essential for the synthesis of neurotransmitter acetylcholine; and the nerve growth factor-tyrosine kinase A receptor is necessary for binding of the neuronal trophic factors, thereby ensuring the survival of the neurons (Johnson et al., 2018; Indo, 2018).

In another experiments, it has been shown that the oral administration of the aqueous extract of the CP roots (150 mg/Kg) to scopolamine induced rats causes a marked reduction in the mRNA levels of tau protein (Bihaqi et al., 2012). Such reduction in the tau protein expression is responsible for causing an amelioration in the amyloid β-induced deficits in case of neurodegenerative disorders such as Alzheimer's disease (Vossel et al., 2010). Hence, the presence of phytoconstituents such as convolvine might be responsible for endowing CP with the abilities to regulate all the neuronal factors/proteins and enzymes, thereby showcasing its evident neuroprotective status (Sethiya et al., 2009; Ray and Ray, 2015).

### Anxiolytic Activity

The ethanolic and chloroform extracts isolated from the aerial parts of CP showed significant anxiolytic activity as recorded using elevated plus maze test on experimental mice. There was increase in the time spent in open arms; and in the number of open arm entries upon the CP oral administration to mice at a dose of 200 mg/Kg (Bhalerao et al., 2014). Similar results have also been observed in case of the ethanolic extract of the CP flower petals at doses 200–400 mg/Kg in mice (Kaushik, 2017).

In another study, CP methanolic extract was evaluated for anxiolytic activity on Obsessive Compulsive Disorders (OCDs) in mice by employing marble burying behaviour analysis, hole board and rota-rod tests. The results have shown that the mice group treated with 200–400 mg/Kg CP methanolic extracts can modulate serotonin or dopaminergic levels, thereby harmonizing the major pathway involving serotonergic or dopaminergic receptors manifesting obsessive compulsive disorders (Subramani et al., 2013). Such anxiolytic activity can be linked with the hypotensive effect of this herb, which in turn is attributed to the presence of GABA-A-benzodiazepine agonists, such as convolamine and scopoletin (**Figure 1B**) (Malik et al., 2011; Amin et al., 2014; Siddiqui et al., 2014).

### Anti-Convulsant Activity

The chloroform, ethanol and aqueous extracts of CP have been evaluated for anti-convulsant activity against strychnine induced as well as pentylene tetrazol (PTZ) induced convulsive seizures in different animal models. Five hundred mg/Kg concentration of the CP extracts have shown statistically significant (p < 0.001) protection against strychnine and PTZ induced clonic convulsions (Ratha and Mishra, 2012; Siddiqui et al., 2014). Methanolic extract of this plant (500–1000 mg/Kg) also exhibited anti-convulsant activity, as characterized by reduction in the mean recovery time of convulsions in case of maximal electroshock seizure model in mice (Kaushik, 2017).

The fundamental mechanism behind such evident anti-convulsing activity of CP might be the presence of coumarins and triterpenoids (Quintans Júnior et al., 2008). Moreover, it has also been proposed that the anti-convulsant activity of a phyto-medicine is escalated by the presence of certain functional groups like, quinoline, quinazoline, thiazole, benzothiazines, oxadiazole, pyridine, pyrazole, imidazole, pyrimidine, phthalazine, triazine, triazoles, cyclopropane carboxylate, and oxime ether (Wei et al., 2015; Song and Deng, 2018). Indeed, many such functional groups have been found in the CP phyto-constituents (**Table 2**) for *e.g.*, quinones, microphyllic acid, 6-methoxy coumarin, 4'-methoxy kaempferol, 7-methoxy quercetin, vanillic acid, syringic acid and melilotic acid (Daniel, 2016).

### Anti-Depressant Activity

Bhalerao and co-workers have found that the chloroform fraction isolated from the CP ethanolic extract reversed the reserpine-induced extension of immobility period of mice in Forced Swim Test (FST), and elicited a significant antidepressant effect by interaction with adrenergic, dopaminergic and serotonergic systems (Bhalerao et al., 2014). Similarly, a polyherbal formulation (Trans-01) containing *C. prostratus* (30%), *Valeriana wallichii* (45%), *Plumbago zeylanica* (7.5%), *Boswellia serrata* (15%) and *Acorus calamus* (3.5%) also exhibited similar anti-depressant properties as tested by employing the forced swim test (FST), tail suspension test (TST) and forced swimming stress (FSS)-induced alterations in serum corticosterone levels. In TST and FST, Trans-01 showed a dose-dependent decrease in immobility time. Moreover, Trans-01 significantly attenuated the elevated corticosteroid levels, thereby indicating a significant anti-depressant activity of this formulation (Shalam et al., 2007). CP herb is known to contain alkaloids (convolamine and scopoletin), flavonoids (kaempferol), and steroids (phytosterol and β-sitosterol). These phytoconstituents most probably act as GABA-A-benzodiazepine agonists and bind to the GABA-A-benzodiazepine receptors, thereby causing an increase in the chloride ion flux and consequent hyperpolarization of the postsynaptic membrane. Such hyperpolarization leads to a hypnotic effect and may alleviate depression (Siddiqui et al., 2014).

### Anti-Inflammatory Activity

Hydroxy-cinnamic acid is a phenyl-propanoid compound found in CP. It is known to cause a downregulation in the expression of cytokine mediators such as IL-8, MCP-1 and ICAM-1, thereby blocking the expression of cytokine-mediated adhesion molecules and therefore the fundamental process of leukocyte–endothelial cell adhesion is deterred (Billore et al., 2005; Rathee et al., 2009). Hence, the CP herb may aid in ameliorating the conditions of neuro-inflammation and consequent cognitive impairment. Indeed, oral administration of the ethanolic extract of the CP leaves at dose of 800 mg/Kg, showed significant inhibition of rat paw edema, in the Carrageenan-induced paw edema and Cotton pellet-induced granuloma animal models (Agarwal et al., 2014).

### Anti-Oxidant Activity

The reactive oxygen species are known to deteriorate the cellular physiology of nerve cells and ultimate lead to neurodegenerative disorders. Polyphenols, flavonoids and vitamin E present in the CP plant act as reactive oxygen species (ROS) scavengers and also ameliorate the lipid peroxidation, thereby attributing towards the anti-oxidant activity of CP (Nasri et al., 2015). It has also been observed that the ethyl acetate and methanolic extract of CP have shown appreciable results (IC_50_ ~ 0.07 mg/mL and 0.075 mg/mL, respectively), as observed in 2,2-diphenyl-1-picrylhydrazyl (DPPH) assay. Similarly, in Ferric Reducing Antioxidant Power (FRAP) analysis, aqueous CP extract has been found to be potentially active with anti-oxidant capacity of 460 ± 8 ascorbic acid equivalent/mg of the extract (Jain et al., 2011). Interestingly, the *Shankhpushpi* syrup and its isolated compounds (Scopoletin and Bacoside A) also exhibited evident anti-oxidant activity as evaluated by using DPPH assay with average IC_50_ value ranging from 0.94 - 2.39% v/v (Jain et al., 2017; Rachitha et al., 2018). Furthermore, the aqueous extract of CP roots diminished the endogenous levels of reactive oxygen species in tauopathy flies as induced by overexpression of τ-protein, thereby substantiating its oxidative stress ameliorative effect (Olakkaran and Antony, 2017).

### Analgesic and Spasmolytic Activity

The ethanolic extract of CP at dose 750 mg/Kg showed statistically significant analgesic activity as compared to the standard analgesics like, morphine sulphate, when tested in hot plate method and tail-flick assays in rats (Agarwal et al., 2014). Such evident analgesic activity is cohesively attributed by flavonoids, volatile oils, alkaloids, polyphenols and organic acids by means of prevention of the formation of cyclooxygenase enzyme and prostaglandins, *i.e.*, mediators of pain sensitization. Hence, these CP phyto-constituents ultimately aid in ameliorating the neuronal pain and headache (Aleebrahim-Dehkordy et al., 2017).

The CP ethanolic extract has exhibited spasmolytic activity in isolated rabbit ileum, isolated rat uterus, intact intestine and tracheal muscles of dog (Barar and Sharma, 1965). Such anti-spasmodic action is linked to the inhibition of acetylcholine production which is mainly brought about by the specific alkaloid present in this CP herb, convolvine (Amin et al., 2014).

### Sedative Activity

Interestingly, the ethanolic and aqueous extracts of the CP aerial parts showed statistical significant potentiation of sleeping time in rats induced with thiopental sodium (Siddiqui et al., 2014). In another similar experiment, the aqueous extract of the CP leaves and flowers showed an evident barbiturate hypnosis potentiation in albino rats at a dose of 300 mg/Kg (Mudgal, 1975). Such sedative activity is directly linked to the presence of phytoconstituents like convolamine and scopoletin which act similarly to GABA-A agonists, thereby bringing about the effects of sedation (**Figure 1B**) (Siddiqui et al., 2014).

This CP herb has also been reported for several other pharmacological activities, including anti-diabetic, anti-hyperlipidemic, anti-hypertensive, anti-microbial, anti-platelet aggregation, anti-ulcer, cardio-vascular, hepatoprotective, and hypothyroidism (Barar and Sharma, 1965; Mudgal, 1975; Rizk et al., 1985; Mali, 1995; Jain et al., 2011; Ravichandra et al., 2013; Jalwal et al., 2016). Anti-diabetic activities of this plant might be attributed to the presence of tropane alkaloids which are known as potent inhibitor of α-glucosidases and R-galactosidases (Gaikwad et al., 2014; Rachitha et al., 2018). The polyphenols present in this species act as reactive oxygen species (ROS) quenchers, thereby ameliorating the oxidative stress that is generated as a diabetic manifestation. Additionally, the presence of vitamin E in this herb also aids in controlling the levels of protein oxidation and lipid peroxidation, thereby leading to an escalation in the antioxidant defense system (Cowan, 1999; Nasri et al., 2015; Domínguez-Avila et al., 2016; Foretz et al., 2018). Furthermore, the presence of GABA-A-benzodiazepine agonists, such as convolamine, scopoletin, ceryl alcohol, kaempferol, phytosterol, and β-sitosterol endow this herb with hypotensive and sedative activities (Malik et al., 2011; Siddiqui et al., 2014). More specifically, a compound, namely, 29-oxodotriacontanol, isolated from the CP herb has also been assessed to possess antimicrobial and anti-fungal activity (Amin et al., 2014). Certain flavonoids and phenyl-propanoids from CP have been shown to provide anti-platelet aggregation and anti-ulcerogenic activity by means of inhibition of cyclic nucleotide phosphodiesterase enzyme and clot retraction capabilities (Beretz and Cazenave, 1991; Tognolini et al., 2006). The anti-ulcerogenic effect was largely observed due to upregulation of mucosal defensive factors such as mucin and glycoprotein secretion, which in turn was induced by the flavonoids and steroids present in this herb (Srinivas et al., 2013). These flavonoids also pose the profound effects on the thyroid hormone regulation and deiodinase-1 inhibition, thereby endowing this herb with anti-thyroid activity (Nagarathna and Jha, 2013). Additionally, the fundamental principle responsible for the cardio-vascular activity of this herb has been proposed to be linked with it alkaloid derivative, evolvine hydrochloride, which is known to exhibit lobeline-like action on the cardiovascular system. This phytoconstituent acts as a cardiac depressant, ultimately leading to a fall in blood pressure, which gets gradually normalised (Dwoskin and Crooks, 2002; Sethiya et al., 2009).

### Safety Profile of *C. prostratus*

The ethanolic and aqueous extracts of the CP leaves have been evaluated for acute oral toxicity study in albino Wistar rats. The animals did not show any toxicity or behavioural changes up to the dose of 5,000 mg/Kg (Agarwal et al., 2014). Similarly, the iron oxide nanoparticles of the CP herb exhibited maximum tolerable dose up to 2,000 mg/Kg in Swiss albino mice with no clinical signs of toxicity. The histopathology of brain also did not show any aberrations or degeneration of neurons. Furthermore, no inflammation was observed in the heart and liver (Ravichandra et al., 2013). These toxicological studies, therefore, confirmed that the administration of CP is safe for the vital organs within the respective treatment durations.

### Summary and Way Forward

The use of herbal medicines continues to escalate rapidly with about 70% of the world population still relying upon traditional medicines for their primary healthcare needs. The natural plant products have negligible toxicities, if any, and are endowed with a multitude of phytoconstituents which are responsible for their holistic therapeutic action. Cognitive dysfunction is one of the major health problem in today's world, wherein the available synthetic chemotherapeutic modalities have proven to be non-absolute and, at times toxic in nature. In such a scenario, safer herbal alternative medicines play a vital role in managing the neurological etiologies. One such cognitive booster herb is *C. prostratus* Forssk., commonly known as *Shankhpushpi*, which is mainly endowed with neuroprotective, nootropic and neuro-modulatory activities (**Figure 1B**). Besides, it also possesses several other therapeutic properties, such as immunomodulatory, antimicrobial, antidiabetic and cardioprotective activities. The fundamental bioactive compounds responsible for the nootropic activities of this herb have been identified as 4'-methoxy kaempferol, 7-methoxy quercetin, convolamine, scopoletin, ceryl alcohol, β-sitosterol and hydroxy-cinnamic acid. Additionally, this herb did not exhibit any signs of toxicity and neurodegeneration up to a dose of 2000 mg/Kg in mice, thereby indicating its safety profile. A few initial clinical trials have conducted for CP, however, more detailed and controlled clinical trials are needed to establish and validate the neuro-pharmacological profile of *C. prostratus*. In addition, detailed mechanistic studies are yet to be executed to unravel the underlying mechanism of action for this cognition enhancing herb. Taken together, *C. prostratus* is likely to be the front runner for the clinical phyto-pharmaceutical status for treatment of neurological ailments.

## Author Contributions

AB conceived the presented research. PT analyzed the information, generated the artwork, and co-wrote the manuscript. AV investigated and supervised the findings of the work. AB and AV provided critical revision of this review article, and approved the manuscript for submission. All authors agreed with the final version of this manuscript.

## Funding

The presented research work been funded by the research funds of Patanjali Research Foundation Trust (PRFT), Haridwar, India.

## Conflict of Interest

The authors declare that the research was conducted in the absence of any commercial or financial relationships that could be construed as a potential conflict of interest.
